# Mutation and selection processes regulating short tandem repeats give rise to genetic and phenotypic diversity across species

**DOI:** 10.1111/jeb.14106

**Published:** 2022-10-26

**Authors:** Max Verbiest, Mikhail Maksimov, Ye Jin, Maria Anisimova, Melissa Gymrek, Tugce Bilgin Sonay

**Affiliations:** ^1^ Institute of Computational Life Sciences, School of Life Sciences and Facility Management Zürich University of Applied Sciences Wädenswil Switzerland; ^2^ Department of Molecular Life Sciences University of Zurich Zurich Switzerland; ^3^ Swiss Institute of Bioinformatics Lausanne Switzerland; ^4^ Department of Computer Science & Engineering University of California San Diego La Jolla California USA; ^5^ Department of Medicine University of California San Diego La Jolla California USA; ^6^ Department of Bioengineering University of California San Diego La Jolla California USA; ^7^ Institute of Ecology, Evolution and Environmental Biology Columbia University New York New York USA

**Keywords:** short tandem repeats, microsatellites, DNA repair, selection, complex traits, evolution

## Abstract

Short tandem repeats (STRs) are units of 1–6 bp that repeat in a tandem fashion in DNA. Along with single nucleotide polymorphisms and large structural variations, they are among the major genomic variants underlying genetic, and likely phenotypic, divergence. STRs experience mutation rates that are orders of magnitude higher than other well‐studied genotypic variants. Frequent copy number changes result in a wide range of alleles, and provide unique opportunities for modulating complex phenotypes through variation in repeat length. While classical studies have identified key roles of individual STR loci, the advent of improved sequencing technology, high‐quality genome assemblies for diverse species, and bioinformatics methods for genome‐wide STR analysis now enable more systematic study of STR variation across wide evolutionary ranges. In this review, we explore mutation and selection processes that affect STR copy number evolution, and how these processes give rise to varying STR patterns both within and across species. Finally, we review recent examples of functional and adaptive changes linked to STRs.

## Graphical abstract

Schematic representation of STR mutations and their possible impact on phenotype. STR mutations frequently arise from misalignment of DNA strands. Strand misalignment may lead to expansions or contractions in repeat copy number depending on which strand the loop forms on (four different mechanisms are represented on top). Many STRs show length‐dependent mutation rates, which can result in bimodal allele length distributions (bottom left). STRs positioned upstream of a gene's transcription start site are depicted at the bottom. Green boxes indicate STR units and black boxes indicate SNPs. The number of green boxes shows the STR copy number of different alleles. This variation serves as the y‐axis of the gene expression graph and the phenotype graph on the right side.

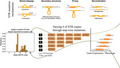

## 1 INTRODUCTION

Short tandem repeats (STRs), consisting of repeated units of 1–6 bp, represent some of the most variable genomic elements. They are found in abundance in genomes of diverse species across the tree of life. The repetitive structure of STRs gives rise to frequent mutations during cell division, making STR loci prone to rapid contractions and expansions. In comparison to point mutations, STRs are 100–10 000 times more mutable (Sun et al., 2012), and often harbor multiple common alleles within a population. Because of their high polymorphism rates, STRs have long been used for a variety of practical applications, including forensics (Ruitberg et al., 2001), paternity testing (Thomson et al., 1999), and linkage analysis in a wide range of species (Ihara et al., 2004; Sheffield et al., 1995). However, it has become clear that these loci are not always neutral and may in some cases play important functional roles.

The high rate of genetic diversity dramatically expands the opportunities for natural selection (Kashi & King, 2006; Nithianantharajah & Hannan, 2007). Indeed, a growing body of literature suggests a key evolutionary role for STRs in shaping phenotypic and genomic diversity within and across species. Classical studies spanning the last three decades have identified important roles of individual STR loci. One example is natural selection on STR alleles in the *period* gene in *Drosophila melanogaster*, where variation in allele length enables balancing fluctuations of the circadian clock according to differing environmental conditions (Sawyer et al., 1997; Zamorzaeva et al., 2005). In another study, it was shown that variation in length of STRs in *Alx‐4* and *Runx‐2* underpin morphological differences across different dog breeds (Fondon & Garner, 2004). Further, comparative genomics studies have demonstrated functional effects of STRs by analyzing patterns of genomic STR conservation and diversification (Karlin & Burge, 1996; Sulovari et al., 2019).

Early studies of evolutionary patterns at STRs were based on individual loci, often relied on limited available datasets, and focused primarily on protein‐coding repeats. Over time, the potential functional role of non‐coding STRs in modulating changes in gene transcription, expression, recombination or chromatin spatial organization has also become increasingly appreciated (Gemayel et al., 2010; Kashi & King, 2006; Vinces et al., 2009). Genome‐wide analyses of patterns of STR variation both within and across species have the potential to reveal novel insights. Yet, STRs have mostly been bypassed in large‐scale evolutionary studies because their highly variable nature complicates their accurate sequencing and genotyping. This changed only recently, with improvements in sequencing technology (Goodwin et al., 2016), increased availability of high‐quality assemblies of diverse species (Rhie et al., 2021) and the development of repeat‐compatible bioinformatics approaches (Dolzhenko et al., 2017, 2019; Highnam et al., 2013; Mousavi et al., 2019; Willems et al., 2017). These developments have allowed for systematic studies of STRs at unprecedented scales across wide evolutionary ranges. This has yielded many interesting findings in recent years, motivating this review of our current understanding of these important but often overlooked genomic elements. We note that mutation and regulatory mechanisms related to pathogenic STR variants specific to humans are reviewed in detail elsewhere (Guo et al., 2017; Massey & Jones, 2018; McGinty & Mirkin, 2018; Mosbach et al., 2019; Neil et al., 2017; Richard, 2021; Wheeler & Dion, 2021; Xiao et al., 2022) and are not the primary topic of this review.

Here, we focus on variation in STR copy number across healthy individuals from diverse species. We begin by reviewing our current understanding of the mechanisms underlying stepwise STR polymorphisms, and how they give rise to patterns of STR genotypes within species. Next, we discuss how these patterns lead to differences in STR characteristics between species and clades over time. Finally, we survey literature examining the phenotypic effects of stepwise STR variation, and how it contributes to shaping complex traits.

## 2 STR VARIATION WITHIN SPECIES

**FIGURE 1:**
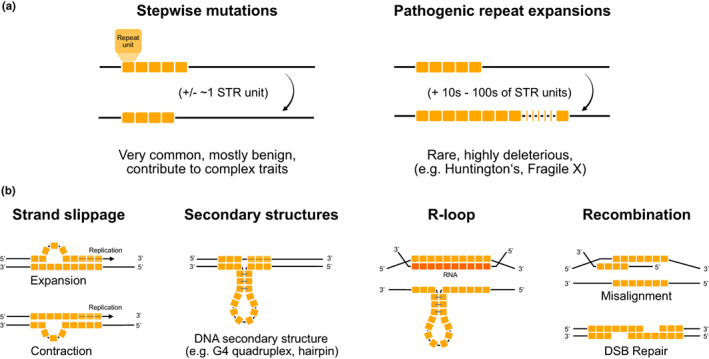
Overview of STR mutations. (a) Patterns of STR mutations. The majority of STR mutations result in small stepwise variation in repeat copy number. These frequent mutations are likely to have little or no phenotypic effects. Larger expansion mutations are rare but may have severe phenotypic consequences in humans, such as in the case of Huntington's Disease, Fragile X Syndrome, or hereditary ataxias (Hannan, 2018). (b) Multiple mechanisms promote STR mutations. STR mutations frequently arise from misalignment of DNA strands. Strand misalignment may lead to expansions or contractions in repeat copy number depending on which strand the loop forms on. Misalignment may happen due to multiple processes including strand slippage (left), formation of secondary structures such as G4 quadruplexes or hairpins during replication (middle left) or as part of R‐loops during transcription (middle right), during homologous recombination, or during repair of double‐stranded breaks (DSB; right).

Short tandem repeats often exhibit high rates of polymorphism within members of a species. This variation arises due to frequent mutations that result primarily in changes in the number of repeat copies. These mutations can take multiple forms. On one hand, very long STRs may become highly unstable, and can result in large repeat expansions, including those involved in disorders such as Huntington's Disease or Fragile X Syndrome in humans. While these mutations are relatively rare, they can have devastating effects. On the other hand, the majority of STR mutations genome‐wide result in modest stepwise changes of +/−1 or more copies of the repeat unit (Figure 1a). These mutations are far more frequent, and most will likely have little or no phenotypic effects. However, increasing evidence points to a widespread role of these stepwise changes in complex traits through regulation of gene expression or other means, which is described in Section 4 below.

### 2.1 Mechanisms leading to STR mutations

Multiple mechanisms contribute to mutations altering STR length (Figure 1b). The majority of STR mutations are thought to arise through “strand slippage,” in which strand misalignment leads to stepwise changes within repeat tracks (Fan & Chu, 2007). Formation of a loop on the extending or template strand leads to expansion or contraction of the newly synthesized DNA molecule, respectively. Slippage can result in STR mutations in the germline leading to inherited variation in STR length. Alternatively, slippage may occur during mitosis of somatic cells, as in the case of microsatellite instability (MSI) in cancer (Hause et al., 2016). This has also been observed in the brain (Kacher et al., 2021) and in other tissues (Breuss et al., 2020). Observations that the majority of STR mutations result in stepwise changes in copy number (Mitra et al., 2021; Sainudiin et al., 2004; Sun et al., 2012) are consistent with polymerase slippage being a major contributor to the STR mutation load.

Several processes can promote strand misalignment in STR regions during replication or transcription and contribute to mutations. For example, some GC‐rich STR sequences can form stable secondary structures such as G4 quadruplexes or hairpins. These structures can result in polymerase stalling, in which the process of DNA replication is impeded, and lead to genome instability (Murat et al., 2020). When these structures occur in transcribed regions, R‐loops, a stable DNA‐RNA hybrid structure, can form. If unresolved, R‐loops can lead to repeat expansions (Lin et al., 2010). These structure‐forming repeats tend to have a lower rate of length‐changing mutations in humans, but are associated with increased rates of point mutations within or nearby the repeat (Murat et al., 2020), which may constrain deleterious expansions at these loci.

Short tandem repeat mutations may also arise through homologous recombination, either during repair of DNA damage from double stranded breaks or unequal crossing over during meiosis (Fan & Chu, 2007). STR mutation mechanisms related to DNA damage may play a more important role in the female germline, since gametes lie dormant for many years during which they may accumulate mutations (Gao et al., 2019). Observations that genome‐wide STR mutations inherited from the maternal germline tend to be slightly larger than paternally‐inherited mutations (Mitra et al., 2021), and that some large repeat expansions are often of maternal origin (Usdin et al., 2015) are consistent with a more dominant role of replication‐independent processes in mutations originating in maternal gametes.

### 2.2 Patterns of STR polymorphism within species

**FIGURE 2:**
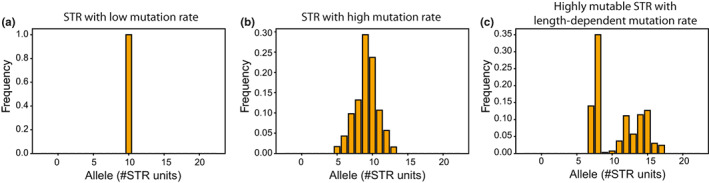
Example simulated allele length distributions for STRs with different mutation properties. Each panel shows allele frequencies at a single STR locus based on a single forward simulation (see Supplementary Methods). (a) Some STRs, such as very short repeats or those with long repeat units (>4bp), have low mutation rates and may not be polymorphic in a population. (b) Repeats with rapid mutation rates may show a wide range of repeat copy numbers. (c) Many STRs show length‐dependent mutation rates, which can result in bimodal allele length distributions.

Short tandem repeats exhibit unique patterns of variation within species compared with other types of genomic elements. They are frequently multi‐allelic. Single nucleotide variants (SNVs) and small indels typically result from a single ancestral mutation resulting in two possible alleles at a single site. In contrast, STRs experience stepwise mutations that affect their length, which is often characterized by recurrent mutations at the same locus. Thus, a single STR locus may display a wide range of possible lengths (alleles) in a population. Although on average STRs are highly polymorphic, there is tremendous variation in mutation rate and patterns, and therefore polymorphism levels across different STR loci (Figure 2).

Multiple factors influence observed patterns of length variation at a particular STR. The *mutation rate* describes the expected rate of mutation at a single locus per generation. On average, STRs experience per‐locus mutation rates that are orders of magnitude higher than those observed for SNVs in humans (Marriage et al., 2009; Sun et al., 2012). However, STR mutation rates can range from <10^−8^ to ~10^−2^ depending on properties of the locus. The strongest and most consistently observed contributor to STR mutation rate is length: STR alleles with the longest stretch of perfect repeats mutate most rapidly, a trend that has been observed across a range of species (Kelkar et al., 2008; Mitra et al., 2021; Payseur et al., 2011; Schug et al., 1998; Sun et al., 2012; Vigouroux et al., 2002; Willems et al., 2016). Repeat unit length is another key determinant of mutation rates. Overall, repeats with shorter repeat units (mononucleotides and dinucleotides) are more variable than longer repeats (Fan & Chu, 2007; Mitra et al., 2021; Payseur et al., 2011). Repeat unit sequence may also play a role: for example AT repeats have been observed to mutate more rapidly than other dinucleotide STRs (Marriage et al., 2009; Sun et al., 2012) and AAAG and AAGG repeats have been shown to be most prone to expansion compared to other tetranucleotide STRs (Bacolla et al., 2008; Kelkar et al., 2008). Mutation rate and heterozygosity have been correlated to a lesser extent with additional genomic features including recombination rate, G/C content, and chromatin accessibility (Fan & Chu, 2007; Mitra et al., 2021; Payseur et al., 2011) although the exact role of these other features in shaping STR mutation remains less clear.

Another feature shaping observed patterns is the *mutation step size*. Most mutations result in changes of a single repeat unit. Multi‐unit changes are possible but their frequency decreases with the size of the step size (Mitra et al., 2021; Sun et al., 2012; Weber & Wong, 1993). STR mutations may result in expansions or contractions, although in humans a slight bias toward expansion mutations has been observed (Ellegren, 2000; Mitra et al., 2021). More specifically, STR mutations have been shown to have a direction bias depending on the size of the parent allele: long alleles tend to experience more frequent contractions, whereas shorter alleles tend to expand. The origins of this direction bias are not well understood and are further discussed in Section 2.4 and the Supplementary Note. Like mutation rate, mutation step size distributions vary widely across different STR loci. For example, dinucleotide STRs tend to have more frequent multi‐unit mutations compared to other STR classes in humans (Mitra et al., 2021; Sun et al., 2012).

The wide variation in mutation rates and step sizes across STR loci (Ellegren, 2000) can give rise to a wide range of patterns of polymorphism in a population. Figure 2 uses a simulation framework (Supplementary Methods in Appendix S1) to explore example distributions of allele sizes for loci with a range of mutation parameters. Loci with very low mutation rates or strong direction bias, such as most trinucleotide repeats in coding regions, will exhibit little to no variation in repeat length (Figure 2a). On the other hand, loci with high mutation rates and modest direction bias, such as long dinucleotide repeats, will show highly variable lengths (Figure 2b). Further, length‐dependent mutation rates can result in bimodal length distributions, in which short alleles remain stable but occasional expansion mutations give rise to a distribution of more mutable longer alleles in the population (Figure 2c).

### 2.3 Genetic determinants of variation in STR mutation within species

The mutation mechanisms described above (Figure 1) are controlled by a complex array of DNA repair proteins related to mismatch repair (MMR) and other processes which are remarkably conserved across species (Li, 2008). MMR is initiated by recognition of replication errors by MutS proteins (MSH2/MSH6 or MSH2/MSH3 heterodimers) and recruitment of MutL (MLH1/PMS2, MLH1/PMS1, or MLH1/MLH3 heterodimers) and other repair proteins (Li, 2008). Inherited genetic variation affecting the function of these proteins can lead to variability in STR mutation rates or properties across individuals of the same species (Usdin et al., 2015).

In humans, germline mutations in key MMR proteins have been shown to modify somatic instability and disease severity of Huntington's Disease and other disorders (Flower et al., 2019; Genetic Modifiers of Huntington's Disease (GeM‐HD) Consortium, 2015; Goold et al., 2021). Similarly, germline mutations in MMR proteins are well known to result in Lynch Syndrome, an inherited condition that predisposes to colorectal and other cancer types with high rates of microsatellite instability (Lynch et al., 2009). Effects of mutations in DNA repair proteins on STR stability in mammalian systems were recently reviewed in detail (Wheeler & Dion, 2021).

Similar effects have been observed in other species. In rice, OsMSH6 mutants showed STR instability at 15/60 STRs analyzed (di, tri, and tetra), as well as an increase in homologous recombination (Jiang et al., 2020). Similarly, suppression of the MMR system induced STR instability (Xu et al., 2012) in rice. In *Caenorhabditis elegans*, *pms‐2* and *mlh‐1* knockouts resulted in a dramatic increase in base substitution and indel rates, in particular 1bp indels at mononucleotide repeats (Meier et al., 2018). *Msh‐2* knockdown was shown to contribute to a ~328x increase in the rate of small indels in *C. elegans* mutation accumulation lines, particularly at mononucleotide runs (Katju et al., 2021).

Beyond genetic determinants, STR mutation patterns may be controlled by environmental effects. In *C. elegans*, it was found that mononucleotide mutation spectra observed in mutation accumulation experiments in a laboratory environment are highly different from those observed from natural variation (Rajaei et al., 2021). In zebrafish, heavy metal exposure and other mutagens have been shown to interact with MMR and promote STR instability (Feitsma et al., 2008; Hsu et al., 2010; Tang et al., 2013; Wu et al., 2017).

### 2.4 Processes constraining STR length

It has long been observed that the length of STRs is constrained ‐ alleles cannot grow to arbitrarily large lengths, and STRs also rarely disappear by contracting to 0 repeat copies. Multiple independent lines of evidence support a model in which STR allele lengths are biased toward staying in a particular range (Bhargava & Fuentes, 2010; Garza et al., 1995; Harr & Schlötterer, 2000; Wierdl et al., 1997). Direct observations of de novo mutations in humans show that long alleles are more likely to contract, whereas short alleles are prone to expansion (Mitra et al., 2021; Sun et al., 2012). Further, in the human population STR allele lengths appear to reach an equilibrium distribution over time (Gymrek et al., 2017; Sun et al., 2012). If STRs mutated by a simple stepwise model with no length constraint, variance would be expected to grow linearly over time. Notably, this trend does not hold true for large unstable repeats such as those implicated in repeat expansion disorders, where large alleles above a certain threshold become highly prone to expansion (Usdin et al., 2015).

Recent evidence suggests that limits on STR length are driven by multiple processes. First, mutation mechanisms may constrain STR length, even at STR loci that are not under natural selection. In this case, allele length biases are driven by biochemical processes that are unrelated to the fitness impact of any particular STR allele. A revised version of the classical generalized stepwise mutation model (GSM) quantifies the magnitude of the length constraint for each STR locus (Gymrek et al., 2017). In this model, mutations are biased to mutate back toward a central (“optimal”) allele length. The farther away an allele is from the optimum, the stronger is the bias to expand or contract toward the optimal length. This model fits well to observed patterns of de novo mutations in humans but does not explain the mechanism for this bias. Implications of this model are described in more detail in the Supplementary Note in Appendix S1.

Early work suggested that one mechanism limiting expansions is the accumulation of point mutations within the repeat sequence, which would tend to break down long alleles over time by limiting the length of perfect STRs (Kruglyak et al., 1998). More recently, a comprehensive analysis of DNA synthesis at all possible STR sequences with 1–6 bp repeat lengths used a high‐throughput primer extension assay to investigate the impact of STR sequence and structure on mutation processes (Murat et al., 2020). This study found that DNA polymerase stalling at structured STRs (those able to form G4 quadruplexes or hairpins) induced point mutations caused by error‐prone synthesis, which in turn reduced the rate of STR expansion. The extent of polymerase stalling at each STR was correlated with length constraints predicted by the modified GSM. Alternatively, multiple studies have also implicated inter‐allelic interactions as an additional potential driver of bias in mutation direction (Amos et al., 2015; Heissl et al., 2019; Mitra et al., 2021). These models are supported by observations that individuals with heterozygous lengths often have higher mutation rates than homozygotes. Although the mechanisms driving mutation through inter‐allelic interactions are still unclear, they could be driven by non‐crossover or gene conversion events mediated by recombination (Heissl et al., 2019). In addition to mutation biases, it is likely that purifying selection against deleterious STR alleles also constrains their length. Selection on STRs is discussed in more detail below in Section 4.

## 3 PATTERNS OF STR ABUNDANCE ACROSS SPECIES

**FIGURE 3:**
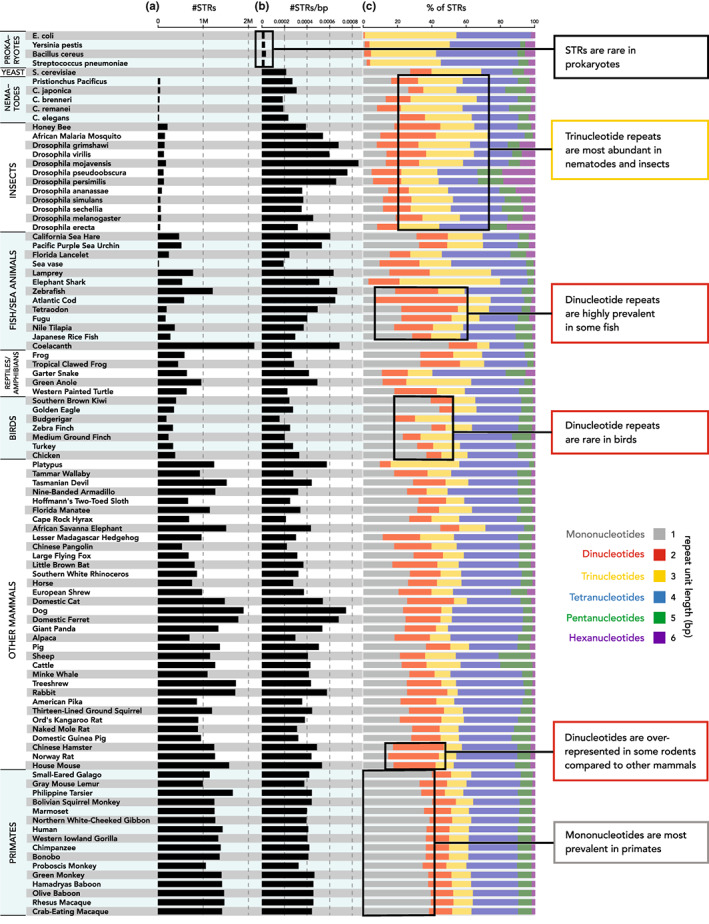
Variability in STR abundance and repeat unit lengths across species. We used Tandem Repeats Finder (Benson, 1999) to detect STRs with repeat units 1‐6bp in genomes from 93 eukaryotic species available from the UCSC Genome Browser (Kent et al., 2002) and 4 prokaryotic species available from NCBI. This analysis is described in more detail in Supplementary Methods. (a) Number of STRs identified per species. (b) STR density (number of STRs divided by genome size). (c) Proportion of STRs by repeat unit length. Gray = mononucleotides; red = dinucleotides; gold = trinucleotides; blue = tetranucleotides; green = pentanucleotides; purple = hexanucleotides. Black boxes highlight specific findings reviewed in the text.

Above, we reviewed mutational mechanisms that give rise to patterns of STR characteristics within species. Over time, these processes will also generate different patterns across different species and clades. This is further compounded by the actions of transposable elements (TEs) (Senft & Macfarlan, 2021; Sulovari et al., 2019) and by natural selection acting on individual STR loci. This section will focus on describing these STR patterns observed across the tree of life. As STRs are much more common in eukaryotes than in prokaryotes (Mrázek et al., 2007; Figure 3), and most studies of STR patterns focus on eukaryotic genomes, the majority of this section will be devoted to eukaryotes. We will briefly discuss STR patterns in prokaryotes and possible explanations for their lower abundance.

Short tandem repeats can be described using various characteristics, the most obvious being the length and sequence of the repeated motif and the number of repetitions. Further criteria are based on the G/C content of the STR and repeat unit purity, i.e., the number of mismatches and indels between units. On a genome‐wide level, the absolute and relative abundance and density of STRs can be quantified. Distilling general patterns of such characteristics from literature is complicated by the use of different datasets, varying STR detection methods and inconsistent definitions of what constitutes an STR (Anisimova et al., 2015; Tørresen et al., 2019). When comparing STRs between species, many authors focus only on perfect repeats, with no mismatches or indels between their constituting units (Mahfooz et al., 2019; Srivastava et al., 2019; Tóth et al., 2000). In other instances, however, imperfect repeats are also considered (Bilgin Sonay et al., 2015; Ding et al., 2017), although accurately annotating these can be challenging (Schaper et al., 2012). Even in the case of perfect repeats, there is still the issue of defining a threshold for distinguishing STR loci from short stretches of repetitive sequence arising by random chance. Some authors define a minimum number of nucleotides that should be part of the repeat region, regardless of motif length (e.g. 12 bp in Srivastava et al. (2019)). Others define a minimum number of repeat units per motif size for a locus to be considered an STR. Such thresholds can be set arbitrarily or estimated through a variety of methods including models based on the occurrence of STRs in genomic sequence (Fondon et al., 2012; Lai & Sun, 2003), *in vitro* experiments (Kelkar et al., 2010) and background occurrences of repetitive tracts in randomly generated DNA (Willems et al., 2014).

### 3.1 STR patterns in eukaryotes

Recent years have seen several comparative genomics studies aimed at investigating patterns of STR characteristics across eukaryotes at various evolutionary distances. From these investigations, we can learn that while it is true that larger genomes tend to have more and longer STRs (Ding et al., 2017; Song et al., 2021; Yuan et al., 2018), the STR density per megabase seems to be rather constant (Srivastava et al., 2019), with some exceptions noted below. Furthermore, G/C content in STRs is generally representative of the corresponding genomic G/C content, which is not correlated to the overall STR density (Srivastava et al., 2019). A study of 719 genomes from various kingdoms (Srivastava et al., 2019) described several taxon‐specific signatures for characteristics such as repeat region length, motif size, as well as G/C content. It reported that the STR density is relatively uniform across eukaryotes, with the main exception being protists. These showed a higher density and a much larger within‐group variation in their STR content compared to other groups. Since the protists are a paraphyly of unrelated primordial eukaryotes, it is not surprising that they display more variability in their STR characteristics. Fungi were found to display the lowest density of STRs, and to have few long STR tracts. Similar findings have been reported in earlier studies of fungal STRs (Dutech et al., 2007).

Another broadly supported observation is that both STR abundance and density are higher in intergenic and intronic regions than in exons (Srivastava et al., 2019; Tóth et al., 2000). The most abundant type of non‐coding STR varies across taxa or species, but usually the abundance patterns are shared within the introns and intergenic regions of one genome. In contrast, all STRs except the tri‐ and hexanucleotide ones are strongly depleted in exonic regions, an observation that holds across the tree of life (O'Dushlaine et al., 2005; Srivastava et al., 2019; Tóth et al., 2000). Because expansions and contractions of coding STRs where the motif size is not a multiple of three will likely result in a frameshift mutation, such repeats are expected to be removed by purifying selection. Coding STRs were also found to be less variable than their intergenic and intronic counterparts (Press et al., 2018), consistent with greater selection constraints on coding sequences.

Interestingly, it was reported that hexamers are the most abundant type of STR in intergenic, intronic and exonic regions in Srivastava et al. (2019). This is in contrast with previous literature, where hexamers were typically found to be among the rarest types of STRs (Ding et al., 2017; Song et al., 2021; Willems et al., 2014). The way in which STR abundances were quantified may be at the root of this apparent discrepancy: Srivastava et al. (2019) compared abundances of different repeat types based on the fraction of all STR bases covered by STRs of a particular motif size. As hexamers have the longest motif size, each hexamer STR unit contributes more to this metric than is the case for other STR types. Long stretches of hexamers such as those making up telomeres, for example, could then substantially inflate the relative abundance of hexamers (Shay & Wright, 2019). We performed a reanalysis of genomes available from the UCSC Genome Browser, which confirms overall clade‐specific trends identified by Srivastava et al. (2019) (Figure 3). Mononucleotide repeats are most prevalent and longest in primates and rarest in insects. Dinucleotide repeats are most prevalent in fish and rarest in birds and are particularly prevalent and long in rodent species. Trinucleotide repeats are relatively rare in mammals and more abundant in fish, insects, and nematodes. Interestingly, tetranucleotides are highly abundant across most taxa. Importantly, we found hexamer repeats to be the least common type of STRs overall. Thus, it appears likely that the high relative abundance of hexamer STRs reported in Srivastava et al. (2019) compared to other sources can be attributed to different quantification approaches.

Another investigation of STRs focusing on 136 insect genomes from various taxa showed that general patterns in STR abundances were relatively well conserved within families of species, but less so on the order level (Ding et al., 2017). This was further demonstrated with a phylogenetic clustering based on the relative abundances of the different STR classes, which could largely recapitulate known phylogenetic relationships between insects. Despite these findings, the authors also report substantial differences in STR characteristics between certain genera and species. As a side note: direct comparisons of genome‐wide STR densities and relative abundances of STR classes in *Drosophila* genomes reported in Srivastava et al. (2019), Ding et al. (2017) and the reanalysis presented in Figure 3 show that while overall patterns tend to be consistent, the absolute values do not always agree. As an example, *D. mojavensis* is consistently reported to have the highest density of STRs among the *Drosophila* species investigated in all three sources, but the absolute value is different in each. This is most likely caused by the differing definitions of what constitutes an STR, as was discussed in the introduction of this section. Another study found that STRs were more abundant in marine fish compared to freshwater fish (Yuan et al., 2018), independent of phylogenetic relationships. This suggests that either the marine environment induced an accumulation of STRs in marine fish, or that fish with a higher proportion of STRs had a selective advantage in this environment. Future studies into the direction of causality for this phenomenon should provide interesting insights into the evolutionary role of STRs.

### 3.2 STR patterns in prokaryotes

Short tandem repeats are less frequent in prokaryotic genomes, with the observed number of perfect STRs often not exceeding the value expected by random chance (Mrázek et al., 2007; Zhou et al., 2014). These findings may be linked to the fact that prokaryotic genomes tend to be composed mostly of protein coding sequences, whereas eukaryotic genomes contain introns and larger proportions of intergenic regions where STRs are more common (Hou & Lin, 2009). This is further supported by the observation that, after correcting for protein length, there is only a relatively small difference in the proportion of tandem repeat‐containing proteins between prokaryotes and eukaryotes (Delucchi et al., 2020). This finding seems to point toward the possibility that the difference in STR abundance between prokaryotes and eukaryotes is caused by the difference in the respective proportions of non‐coding genomic sequences.

While it is true that prokaryotic genomes have fewer STRs compared to eukaryotes, there are subsets of host‐adapted prokaryotes with high STR abundance. A comparison of STRs across 378 prokaryotic genomes (Mrázek et al., 2007) reported that host‐adapted pathogenic bacteria have many long stretches of STRs with motif sizes of 1–4 bp. More recently, similar observations were made for phytopathogenic bacteria (Mahfooz et al., 2019). Both examples involve pathogenic communities of bacteria that are in contact with the host immune system. These pathogens typically exist as heterogeneous populations with different phenotypes. This makes the community more robust to fluctuations in environmental factors and harder to target by the host immune system. Through their high mutation rates, STRs offer an ideal avenue to generate a diverse range of phenotypes rapidly and reversibly within a microbial community. This ‘on/off’ switching of genes is a process termed phase variation, which is reviewed in (Moxon et al., 2006). Section 4 of this review will discuss such phenotypic effects mediated by STR variation more in depth.

## 4 PHENOTYPIC DIVERSITY CREATED BY STRS WITHIN AND BETWEEN SPECIES

Previous sections focused on the highly variable nature of STRs and processes driving this variability. In this section, we review the consequences of genetic variation at STRs on phenotypic diversity with an emphasis on their functional and adaptive impact. In addition, we discuss efforts to develop statistical tests to detect natural selection acting on STRs.

**FIGURE 4:**
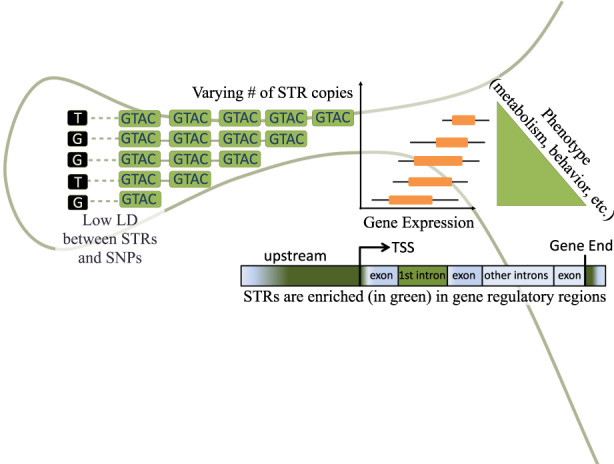
Schematic representation of an association between the length of an STR and a quantitative phenotype. Repeats positioned upstream of a gene's transcription start site are depicted. Left: Green boxes indicate STR units and black boxes indicate SNPs. The number of green boxes shows the STR copy number of different alleles. This variation serves as the y‐axis of the gene expression graph and the phenotype graph on the right side. Variation in STRs is often in low linkage disequilibrium (LD) with nearby SNPs (Jakubosky et al., 2020; Saini et al., 2018). The bar at the bottom right depicts enrichment of STRs in gene regulatory regions as documented in Sawaya et al. (2013) with increased abundance in upstream regions and immediately downstream of genes as well as in first introns (Bilgin Sonay et al., 2015).

The role of STRs in creating genotypic diversity has been well documented in classical population genetics studies (King & Motulsky, 2002; Rosenberg et al., 2002; Slatkin, 1995). Yet, apart from well‐known STRs implicated in repeat expansion disorders, the impact of this variability on phenotype has become a question of interest only in the last two decades. In a seminal chapter in 1999 on variation and fidelity, King and Soller (1999) described STRs as a source of evolutionarily beneficial mutations and suggested they may evolve under positive selection (King & Soller, 1999). Intriguingly, a single STR locus may exhibit an entire spectrum of alleles (Figure 2), allowing it to act more like a tuning‐knob to “adjust” phenotypes rather than a switch (Figure 4). While earlier research has focused on individual locus‐trait links (for example reviewed in Gemayel et al. (2010); Kashi and King (2006)) advances in sequencing, bioinformatics analysis, and population genomic approaches have enabled recent studies to uncover associations at much larger scales (Fotsing et al., 2019; Press et al., 2018; Quilez et al., 2016). With thousands of newly discovered STR‐phenotype associations, developing a standardized methodology to systematically identify STRs under selection and infer their evolutionary fitness impacts remains a frontier in the field.

### 4.1 Protein‐coding STRs

As mentioned in the previous section, STRs in coding regions are less abundant and consist mostly of in‐frame repetitions of tri‐ or hexanucleotide motifs (O'Dushlaine et al., 2005; Srivastava et al., 2019; Tóth et al., 2000). Once transcribed and translated, such STRs result in homorepeats and dipeptide repeats in a protein sequence, respectively. Considering their inherent variability, it is not surprising that even in‐frame STRs in coding sequences are generally avoided as they can affect protein structure and function. The numerous neurodegenerative diseases caused by aggregation of proteins with pathologically expanded repeat tracts ‐ particularly polyA and polyQ ‐ serve as grim examples of the harmful capabilities of coding STRs (Darling & Uversky, 2017). Additionally, several cancers are associated with an increase in STR mutations in their exons (McIver et al., 2014; Sonay et al., 2015). Moreover, STR‐containing proteins are known to have a higher dosage‐sensitivity than non‐STR proteins, i.e. overexpression of STR proteins can have cytotoxic effects (Chavali et al., 2017). There appear to be precautions in place to limit these harmful effects: STR‐containing proteins were demonstrated to be under more stringent proteostatic control than non‐STR proteins, causing lower concentrations and higher turnover rates of such proteins (Chavali et al., 2017). In summary, protein‐coding STRs are a risk to cells, and costly control mechanisms are needed to mitigate their harmfulness. There must, therefore, be a sufficiently important functional niche filled by protein‐coding STRs that makes them evolutionarily beneficial.

The first well‐studied example of a protein‐coding STR variation that is not disruptive but functional comes from *D. melanogaster* studies (Sawyer et al., 1997). An STR copy number mutation acts as a switch mechanism for the length of the circadian rhythm adapting it to the climate. Many additional examples were subsequently discovered, including STRs affecting cell surface variability, skeletal morphology, and other phenotypes in a range of species. These early discoveries of adaptive roles for STR mutations are reviewed in detail in Gemayel et al. (2010). A recent review (Newton & Pask, 2020) expanded on the well‐known example of RUNX2, which has an STR whose glutamine‐to‐alanine residues ratio correlates with cranial skeletal features (first shown in dogs; Fondon & Garner, 2004). The review investigates several taxa and paralogs of the gene to reveal that the repeat, absent in the RUNX1 and RUNX3 homologues, has likely played a crucial role to fine‐tune osteogenesis across vertebrates. Other recent studies also provide ample evidence of functional STR regions: a polyglutamine tract in ELF3 in *Arabidopsis thaliana* was shown to result in reciprocal incompatibilities across two divergent genetic backgrounds, potentially through mediating complex epistatic interactions with other genes (Press & Queitsch, 2017). A deletion at an STR in *PIEZO1* common in African populations results in a gain‐of‐function allele that may provide malaria resistance in humans (Ma et al., 2018). In yeast and humans, proteins with long polyQ stretches were found to have more protein‐protein interactions on average than those containing short or no polyQ tracts. This suggests that variations in the length of polyQ‐encoding STRs may modulate the degree to which proteins are able to form interactions (Schaefer et al., 2012). A peculiar example from Dictyostelid amoebae indicates the functional importance of an extremely long and conserved serine repeat, which is supported by high codon diversity and length variation patterns across paralogous genes and across other species (Tian et al., 2014). Across 153 genomes from the Euarchontoglires superorder, genes with STRs in their coding sequences were consistently enriched with binding‐ and transcription factor‐related functional terms (Song et al., 2021). Zinc‐finger and forkhead box transcription factors in particular were found to harbor many coding STRs. This is consistent with cross‐species protein‐level analysis that reported enrichment of tandem repeats in zinc‐finger proteins (Delucchi et al., 2020).

In many cases the mechanism by which an STR affects protein function remains unclear. However, one clue may be the link between STRs and protein structure. There has been extensive evidence that protein‐coding STRs primarily encode disorder‐inducing amino acids and reside mainly in intrinsically disordered regions (Delucchi et al., 2020; Jorda et al., 2010; Verbiest et al., 2021). This combination of intrinsic disorder and repeating sequence gives rise to proteins that are flexible and can bind to a variety of substrates, be they nucleotide, lipid or protein (Uversky, 2013). Such STR‐containing proteins are involved with regulation of gene expression (Song et al., 2021; Verbiest et al., 2021) and are often pleiotropic and multifunctional, making cells more robust to environmental perturbations (Chavali et al., 2017). Furthermore, they often appear as hubs in protein interaction networks and have interactions with numerous other proteins (Haynes et al., 2006; Press & Queitsch, 2017; Schaefer et al., 2012). These characteristics are evidently indispensable for normal cellular function, and are thus worth the risk incurred by having highly mutable STRs in proteins. To better understand this tradeoff, future investigations should take an evolutionary perspective on protein‐coding STRs, and compare them across species. To this end, servers such as dAPE (Mier & Andrade‐Navarro, 2017) may offer valuable insights.

### 4.2 Non‐coding STRs

Non‐coding STRs are known to affect a variety of phenotypes. Initial studies identified functional STRs, mostly located in known gene regulatory regions. For example, in tilapia fish, STR length in the promoter of the *prolactin 1* gene correlates with the gene's expression and fish mass, and the direction of effect changes with water salinity levels (Streelman & Kocher, 2002). In 2005, Hammock and Young discovered an STR mutation in the 5′ UTR of the *vasopressin 1a* receptor gene that changes social behavior of voles (Hammock & Young, 2005). This study was the first to provide experimental evidence for a clear link between an STR and a consequential phenotypic change. This was followed by another piece of experimental evidence revealing the adaptive role of a tandem repeat‐phenotype link in yeast (Vinces et al., 2009). The authors found that copy number of a tandem repeat in the promoter region of *SDT1* gene correlated with the gene's expression levels and increased growth due to the high expression of *SDT1*, a clear example for what is presented in Figure 4. They could also show that under an experimental evolution setup, repeat copy number evolved to the exact number that achieves the highest gene expression. This study served as the first experimental evidence that tandem repeats evolve under positive selection. Additional early examples of non‐coding STRs are reviewed in (Gemayel et al., 2010).

Genome‐wide studies in multiple species have since revealed a widespread role of non‐coding STRs in regulating gene expression. Yeast was the first species where it was shown systematically that the presence of tandem repeats in gene promoters is correlated with increased gene expression divergence (Vinces et al., 2009). Abundance of STRs in human gene regulatory regions (Sawaya et al., 2013) raised the question of whether such a correlation may hold also in the human genome. Indeed, genes with tandem repeats in their regulatory regions exhibit greater gene expression divergence between humans and other primates, underlining the contribution of these repeats in creating phenotypic diversity (Bilgin Sonay et al., 2015). STRs are estimated to contribute to 10%–15% of heritable variation in gene expression in humans due to common *cis* variants according to the findings in Gymrek et al. (2016). In the same study, the authors identified 2060 STRs showing significant associations with gene expression changes. The number of these so‐called ‘expression short tandem repeats’ (eSTRs) later expanded to more than 28 000 eSTRs by analyzing 17 human tissue types (Fotsing et al., 2019), although only a subset of these are likely to be causally affecting gene expression. Further, a genomic survey on STRs in human populations (Kinney et al., 2019) identified eSTRs whose frequencies significantly differ between ethnicities. The same group also found 15 eSTRs whose repeat length correlates with gene expression (Kinney et al., 2021).

Non‐coding STRs are diverse, ranging from highly unstable mononucleotide repeats to less variable hexanucleotide repeats. This diverse set of repeats likely influences phenotypes through a variety of mechanisms. Studies in yeast have demonstrated that mononucleotide repeats (poly(dA:dT) tracts) act as strong nucleosome positioning signals (Suter et al., 2000) that affect expression of nearby genes when manipulated (Raveh‐Sadka et al., 2012). In some cases, STRs may serve as binding sites for transcription factors. In these cases, altering the number of STR units can modify binding affinity of the transcription factor to DNA. For example, MeCP2, a methyl‐CpG‐binding protein implicated in Rett Syndrome in humans, was shown to bind hydroxymethylated CA repeats in mice in a repeat‐length dependent manner (Ibrahim et al., 2021). In another example, the aberrant EWSR1‐FLI1 fusion protein formed in Ewing Sarcoma binds to GGAA repeats (Gangwal et al., 2008), converting them into de novo enhancers whose length is dependent on the length of the GGAA repeat tract (Riggi et al., 2014). In addition to the STR itself forming a binding site, an in vitro protein‐binding assay showed that STRs may influence the binding affinity of proteins to nearby DNA‐binding sites (Afek et al., 2014). Additional studies have demonstrated that STRs may affect 3D chromatin structure (Sun et al., 2018) and are enriched in chromatin loops (Jakubosky et al., 2020), suggesting a role of non‐coding STRs in epigenetic regulation. A genome‐wide survey in humans revealed that STRs modify gene expression by regulating methylation levels of adjacent genes (Quilez et al., 2016). Other studies have identified over 100 non‐coding STRs as modifiers of DNA methylation (Garg et al., 2020; Quilez et al., 2016). Finally, STRs have been implicated in spacing between regulatory elements, selection of transcription start and termination sites, and alternative splicing in eukaryotic genomes (Bagshaw, 2017).

### 4.3 Detection of STRs under selection in genome‐wide studies

Given their widespread impact on phenotype, STRs are likely to be frequent targets of natural selection. Indeed, recent years have seen several examples of studies where STRs in genes with known adaptive functions were investigated using genome‐wide surveys. In cattle, STRs in genes involved in milk production and fertility were found to be under selection, in line with the recent artificial selection history of the species (Xu et al., 2017). In pigs, a number of polymorphic STRs were associated with temperature and altitude, suggesting that they evolve under selection (Wu et al., 2021). Polymorphic STRs were also found to allow for better identification of pig breeds in comparison to SNPs. Another study in wild orangutan populations identified distinct STR length changes in genes linked to species' recent local adaptations, such as increased brain size and reproductivity (Voicu et al., 2021).

To obtain a more systematic view on the functional and adaptive roles of STRs, genome‐wide scans for natural selection are needed. Traditional genome‐wide scans for adaptive natural selection (Przeworski et al., 2005) are typically focused on SNPs (Press et al., 2019) and their associated haplotypes. However, high rates of STR length changes and frequent recurrent mutations can result in the same adaptive allele on different haplotypic backgrounds (Haasl & Payseur, 2016). These types of ‘soft sweeps’ substantially reduce the power of generic statistics used for detecting regions under positive selection (Press et al., 2019). As a consequence, selection on STRs cannot be reliably detected by these methods.

Several key studies by Haasl & Payseur and colleagues have enabled the first genome‐wide STR selection scans (Haasl et al., 2014; Haasl & Payseur, 2013). The authors presented a novel framework to model the fitness surface of an individual STR that considers unique characteristics known to govern STR genotype‐phenotype relationships (Haasl & Payseur, 2013). They additionally developed a computationally efficient method to simulate allele frequencies at a single STR over time based on theoretically computed allele‐size specific mutation rates. Using their simulation framework, one can infer selection parameters by comparing simulated vs. observed allele frequencies in a population. This method was applied to investigate the origins of Friedreich's ataxia, a heritable disease caused by an STR expansion in the first intron of the *frataxin* gene. In addition, simulations showed that selection on STRs leaves a unique footprint on the site frequency spectrum of neighboring genomic areas that is comparable to soft sweeps on SNPs (Haasl & Payseur, 2013). The inability of generic methods to detect selection on STRs was confirmed in humans, and a novel statistic based on the number of haplotypes and segregating sites was proposed in Haasl et al. (2014). Using this statistic, genome‐wide scans were performed that detected known and novel autosomal STR loci under selection, including a long intronic CA repeat in *MAGI2* (Haasl et al., 2014).

In addition to providing a source of adaptive genetic variation, mutations at STRs may in some cases be deleterious and subject to negative selection. SISTR (Mitra et al., 2021) is a method to measure negative selection individually at each STR in the genome. SISTR models a single selection coefficient (s) at each STR. It assumes an optimal allele length with fitness 1, and that alternate alleles have fitnesses decreasing as a function of s and their distance from the optimal allele. It then uses a simulation framework based on that of Haasl and Payseur (2013) to infer locus‐specific values for s. SISTR was applied to infer selection coefficients based on a panel of STR genotypes obtained from the Simons Simplex Collection. STR mutations estimated to be under the strongest selective pressure were over‐represented in children affected by autism compared to their unaffected siblings.

Further considerations specific to protein‐coding STRs may include analyzing codon purity of STRs or their relationship to differential splicing patterns. For example, negative selection can be detected through high codon diversity in otherwise conserved STRs with distinct variability across homologous proteins, either by using codon diversity statistics (Haerty & Golding, 2010; Tian et al., 2014), or by modeling the evolution of synonymous codons (Huntley & Golding, 2006). Alternatively, deviations from expectations can be determined by comparison to repeat sequences generated under a neutral model (Mularoni et al., 2010). In eukaryotes, characterizing STR variation as a function of exon splicing (constitutive vs alternative) presents another possibility to test for selection on STR and codon diversity, as reported for homopolymer sequences (Haerty & Golding, 2010).

Despite the promising results of studies of the role of natural selection acting on STRs, a consensus on benchmark selection tests and mutation models for STRs still remains to be established. Addressing the challenges that are mentioned above would lead to a more complete understanding of genomic variants that underlie adaptations or are conserved by purifying selection.

### 4.4 Linking STR variation and complex traits

Increasing evidence supports a widespread role for STRs in modulating a variety of traits across diverse species. Press et al. (2014) argued that STR variation likely accounts for a significant portion of the heritability of complex traits in humans and model organisms that is not due to SNPs (Press et al., 2014). However, due to unique challenges STRs pose in genotyping and downstream genomic analyses, the effects of STRs on complex traits remain understudied systematically at a genomic scale.

A major challenge is that many commonly used genome‐wide analysis pipelines do not directly handle STRs. These pipelines are often built to analyze bi‐allelic SNPs, rather than highly multi‐allelic variation in length at STRs. Unfortunately, polymorphic STRs are often only in modest linkage disequilibrium (LD) with nearby SNPs, and thus their effects cannot be fully captured by SNP analysis alone (Figure 4). Indeed, a study in humans combined STRs with structural variants to assess their links to Genome Wide Association Study (GWAS) traits and found that only 11% were tagged by SNPs (Jakubosky et al., 2020). Similar findings were reported for other species, where a significant portion of STRs detected in cattle (Xu et al., 2017) and *Arabidopsis thaliana* (Press et al., 2018) were not tagged by SNPs.

While the past decade has seen the development of increasingly accurate and comprehensive methods for the genotyping of STRs from genomic sequencing data (Dolzhenko et al., 2019; Highnam et al., 2013; Mousavi et al., 2019; Willems et al., 2017), STRs have yet to be fully integrated into genome‐wide studies. As methods for STR analysis continue to improve, we hypothesize that evolutionary roles for STRs will continue to be uncovered and will fill an important gap in the genetics of complex traits.

## 5 CONCLUSIONS

Short tandem repeats are one of the richest sources of genotypic variation, but were long under‐investigated due to technical challenges. They are perhaps most notorious as the drivers of neurodegenerative repeat expansion diseases such as Huntington's disease. However, we now know that they are also involved in many complex traits through stepwise mutation patterns. Recent years have seen an increasing understanding of the mechanisms that govern such STR variability. Here, we reviewed recent insights relating to the processes leading to STR variation and to constraints on the length of STR tracts. While in this review we focused on STRs, similar trends have been observed for longer repeats such as variable number tandem repeats (VNTRs), which also tend to be multi‐allelic and can regulate phenotypes in a dynamic manner (Mukamel et al., 2021; Utgés et al., 2021; Xu et al., 2016).

Frequent mutations in STR loci generate genome‐wide patterns within species that can be used to characterize populations and determine geographical origins of individuals. These mutations ‐ along with other processes ‐ are also responsible for the emergence of different STR patterns across the tree of life. Modern sequencing and bioinformatics methods now allow us to investigate STRs across a wide range of genomes. Recent comparative genomics studies have started to uncover the spectrum of evolutionary STR patterns over time.

The emergence of such patterns is by no means a neutral process, as STR variation can have phenotypic consequences. Protein‐coding STRs can act like an ‘on/off switch’, as is the case in prokaryotic phase variation, or have more subtle effects through the regulation of protein structure and interactions. Non‐coding STRs in regulatory regions can affect phenotype as well, although the effect here is often akin to a ‘tuning‐knob’ where variation in STR length regulates gene expression or other molecular phenotypes. Like any genomic feature affecting phenotype, such STRs are expected to be under natural selection. However, detecting natural selection on STR loci is complicated by their inherent variability. Standard selection detection methods are geared towards SNPs, whereas the high mutability of STRs leads to violations of core statistical models. Here, we have highlighted past and current efforts to develop sound methods for detecting natural selection on STR loci and presented early indications that a large portion of STRs are indeed under selection.

As our capacity to analyze STRs increases, so does our appreciation of the diverse roles these genomic elements play. The ever‐growing availability of sequencing data from different organisms will deepen our understanding of the patterns that these loci form within and across species. Future developments of specialized and standardized approaches to detect natural selection on STRs could position us to unravel their phenotypic effects on a genome‐wide scale across evolution.

## AUTHOR CONTRIBUTIONS

M.V., M.A., M.G., and T.B.S. were involved in planning, orchestrating and writing of the entire manuscript. M.M., and Y.J. contributed to writing and making of the figures in Sections 2 and 3.

## CONFLICT OF INTEREST

The authors have no conflict of interest to declare.

### PEER REVIEW

The peer review history for this article is available at https://publons.com/publon/10.1111/jeb.14106.

## Supporting information


Appendix S1

## Data Availability

Data associated with Figure 3 is available on Figshare (https://doi.org/10.6084/m9.figshare.20520723.v2). Code used for analyses shown in Figure 2 and 3 is available at https://github.com/gymreklab/STR‐Evolution‐Review/ (doi: 10.5281/zenodo.7011508).
