# Supplementary material for: Mutation and selection processes regulating short tandem repeats give rise to genetic and phenotypic diversity across species
Source: J Evol Biol. 2022 Oct 26;36(2):321–36. doi: 10.1111/jeb.14106 (PMC9990875; doi:10.1111/jeb.14106)
Supplement: Supplementary file 1 — Appendix S1 [file JEB-36-321-s001.docx]

**Supplementary Material - Verbiest, *et al.***

## Supplementary Methods

### Simulating TR allele frequencies

Allele frequencies shown in **Fig. 2** were simulated according to a mutation model modified from the length-constrained model described in Gymrek et al. (2017) and using a simulation framework used in Mitra et al. (2021) based on the method originally described in Haasl & Payseur (2013). The model is described in the **Supplementary Note** below. For all panels of **Fig. 2**, we set: length constraint $\beta$=0.1, effective population size=10,000, number of simulation generations=50,000, number of sampled alleles=1,000, and number of possible alleles=25. **Fig. 2a** was generated using a constant mutation rate of $\mu=10^{-8}$ mutations/generation. **Fig. 2b** was generated using a constant mutation rate of $\mu=10^{-3}$ mutations/generation. In both **Fig. 2a** and **2b** we set the step size parameter $\rho=0.9$. **Fig. 2c** was generated using a length-dependent mutation rate, in which the central allele has mutation rate$\mu=10^{-3}$ and the log10 mutation rate scales linearly with the number of units away from the central allele (slope L=0.8). For **Fig. 2c** we set the step size parameter $\rho=0.8$. For all panels we arbitrarily assume a central allele length of 10 copies of the repeat unit.

Code for performing simulations can be found at https://github.com/gymreklab/STR-Evolution-Review.

### Generating genome-wide TR databases for each species

We used Tandem Repeats Finder (Benson, 1999) version 4.09 with parameters “2 5 17 80 10 24 20” to detect short tandem repeats with repeat units 1-20bp in genomes from 93 eukaryotic species available from the UCSC Genome Browser (Kent et al., 2002) and 4 prokaryotic species available from NCBI. Subsequent analysis focused on STRs with repeat units of 1-6bp. STRs were filtered to exclude imperfect repeats following a process similar to that used in GangSTR (Mousavi et al., 2019). The resulting STR regions were further filtered to exclude those with repeat length below 10 (for repeat units 1-2bp), 12 (for repeat units 3-4bp), 15 (for repeat units of 5bp) or 18 (for repeat units of 6bp) total base pairs. We additionally filtered imperfect repeats, and repeat bundles, defined as multiple repeats within close proximity. The scripts used to perform statistical analysis and generate the figure can be found in https://github.com/gymreklab/STR-Evolution-Review.

## Supplementary Note

### Modeling STR mutation dynamics

For our simulation analyses, we use a mutation model based on a classical simple stepwise model (Sainudiin et al., 2004) generalized to multi-unit step sizes, but with two key modifications:

- Length-dependent mutation rate: Studies across a range of taxa have shown that STR mutation rates increase with repeat length [(Kelkar et al., 2008; Payseur et al., 2011; Schug et al., 1998; Sun et al., 2012; Vigouroux et al., 2002; Willems et al., 2016)](https://paperpile.com/c/1zjYAv/8H0V+JwG0+wLaT+NU7C+tVLn+VIzu). Thus, we model allele-specific mutation rates which scale as a function of repeat length.
- Length-biased mutation direction: Multiple studies (primarily based on human data) have identified a length bias [(Bhargava & Fuentes, 2010; Garza et al., 1995; Harr & Schlötterer, 2000; Wierdl et al., 1997)](https://paperpile.com/c/1zjYAv/0fSXa+qOybe+CGo5S+bv2Cv), in which short alleles tend to expand and long alleles tend to contract. To account for this in our model, we assume there is a central (“optimal”) allele at each locus. In our simulations, alleles are scaled in terms of the number of repeat units away from the central allele, with the central allele itself set to 0. Mutations are biased to expand or contract back toward this central allele.

Our mutation model consists of four parameters at each STR locus: $\mu_{0}$(mutation rate of the central allele), $L$ (controls dependence of mutation rate on allele length), $\rho$ (step size parameter), and $\beta$ (length constraint). The probability of transitioning from allele $a_{t}=g$ to $a_{t+1}=h$ in a single generation is given by:

$Pr(a_{t+1}=h|a_{t}=g) = 1-\mu_{g}; h=g$

$= \mu_{g}uP_{X}(h-g); h>g$

$= \mu_{g}dP_{X}(g-h); h<g$

Where:

- $\mu_{g}$ gives the mutation rate of allele $g$. At each generation, mutations occur at a rate dependent on the length of the parent allele. $\mu_{0}$gives the length of the central allele, and the mutation rate $\mu_{g}$ for a parental allele $g$ is set to $10^{{log}_{10}(\mu_{0})+Lg}$.
- $P_{X}$ describes the step size distribution of mutations. $P_{X}(k)$ gives the probability of a mutation resulting in a change in $k$ repeat units and is computed using the probability mass function of a geometric distribution with parameter $\rho$: $P_{X}(k) = \rho(1-\rho)^{k-1}$. $\rho$ controls the size of mutations. If it is set to 1, all mutations are by a single unit. Smaller values for $\rho$ result in more multi-unit mutations.
- $u$ and $d$ give the probability of a mutation resulting in an expansion or contraction in repeat unit number. $u=\frac{1-\beta\rho g}{2}$, $d=\frac{1+\beta\rho g}{2}$, and $u+d=1$. The parameter $\beta$ describes the strength of the length bias in mutation rate. If $\beta$=0, this reverts to a simple stepwise model, where expansions and contractions are equally likely. If $\beta$>0, alleles smaller than the central allele ($g<0$) are biased toward expansions, and alleles larger than the central allele ($g>0$) are biased toward contractions.

### Patterns of STR allele frequencies vary with mutation parameter settings

Simulating allele frequencies under a specified mutation model enables exploring the impact of various mutation parameters on the distribution of allele frequencies at an individual STR locus in a population. We comment generally on the effect of the four parameters of our model below:

- Mutation rate ($\mu_{0}$): as expected, at loci with very low mutation rates, an STR may be fixed at a single allele length in all individuals in the population, whereas at higher mutation rates greater variability is observed.
- Length constraint ($\beta$): if there is a non-zero length constraint ($\beta$>0), and sufficiently high mutation rate, allelic distributions will tend to be unimodal and centered at the central allele (0). On the other hand, in the case of no length constraint, the mode of the distribution may shift away from 0, and in some cases may be multi-modal.
- Step size parameter ($\rho$): Values of $\rho$ less than 1 result in more multi-unit step sizes, and generally more variable allelic distributions.
- Length-dependence of mutation rate ($L$): Length-dependent mutation rates ($L$>0) can lead to more unpredictable allelic distributions. For example, an early mutation leading to a short allele may result in a distribution centered at a stable allele shorter than the central allele. Alternatively, a rare expansion event can create a longer, more mutable allele. This can eventually result in bimodal allelic distributions, where shorter alleles are generally not that variable, but the longer alleles show quite a bit of polymorphism in length.

Example simulated allelic distributions under different conditions are shown in **Fig. 2** in the main text. Additional examples resulting from varying each of these parameters are shown in **Supplementary Figs. 1-4**.

We expect the values of these parameters to be highly variable across STR loci as a function of repeat unit sequence and other features such as genomic context. Further, observed allelic distributions are also subject to random fluctuations due to genetic drift and sampling biases. Thus even for loci with identical mutation parameters, a wide range of possible allelic distributions can often be obtained.

### Limitations of modeling an optimal allele

While multiple studies have suggested STR mutations show a length bias, modeling such a bias poses important challenges. An important question is what biological mechanism could be driving a bias for shorter alleles to expand and larger alleles to contract. Relatedly, what determines the length of the central, or optimal, allele? Multiple plausible hypotheses exist:

(1) The optimal allele could be determined based on biochemical properties specific to DNA replication and repair at each STR locus. In this case, the optimal allele would be expected to be constant across different populations of the same species.

(2) The optimal allele may confer the greatest fitness, with other alleles being deleterious. In this case, the optimal allele could be constant across populations, or could vary based on interaction with environmental effects. While there are likely examples where this is true, we expect stepwise mutations at the majority of STRs to have little or no effect, and that fitness effects alone are unlikely to completely explain the observed length bias.

(3) The length-bias could result from interaction between heterozygous alleles in a parent (Amos et al., 2015; Heissl et al., 2019). If mutations tended to result in repeat lengths intermediate in length between the two parental alleles, allele lengths might tend to regress toward the mean length in the population. It remains unclear what biological mechanism might drive this, since most STR mutations are thought to arise through strand slippage which should not be affected by allelic interaction. Notably, this could lead to different central alleles in different populations, and could also result in the central allele changing over time. This would also imply that mutations arising from homozygous parents, or at loci with lower overall heterozygosity, would not be prone to this length bias.

(4) Finally, the observed bias could arise due to technical artifacts in STR analysis. One source of error in STR genotyping is heterozygous dropout of long alleles. Because long alleles are harder to amplify, and also harder to span with short sequencing reads, individuals who are heterozygous for a short and long allele may tend to be genotyped incorrectly as homozygous for the shorter allele. This type of error pattern could give rise to biases in observed mutation direction. However, the fact that this trend has been observed across multiple studies (Bhargava & Fuentes, 2010; Garza et al., 1995; Harr & Schlötterer, 2000; Mitra et al., 2021; Wierdl et al., 1997) using different technologies (both capillary electrophoresis and whole genome sequencing) and that data from both directly observed de novo mutations as well as across much deeper time scales (Garza et al., 1995; Gymrek et al., 2017; Sun et al., 2012) fit best to a length-constrained model, it is unlikely this trend is entirely driven by technical artifacts.

Understanding the source of this length bias has important implications for improving models of STR mutation and selection. First, our current models assume a constant optimal allele length. However, depending on the biological mechanism driving this bias, models allowing the optimum to change over time might be more appropriate. Second, a related challenge is that the optimal allele at each locus is unknown. A reasonable guess might be the mean or modal allele length in a population. However, mutation simulations show these may not actually be equal to the simulated central allele, in which case these guesses are incorrect. Third, disentangling length-bias from fitness effects remains challenging. We recently developed a method, SISTR (Mitra et al., 2021), in which we model the fitness of each allele as a function of its distance from an optimal allele length. This method requires a neutral mutation model as input, in which we model length constraint toward a central allele using the model above. For simplicity, we assume there that the central allele and optimal allele are identical. However, it is not obvious that this must be the case. Better understanding of the length bias would enable relaxing that assumption and likely result in better inference of selection at STRs. Fourth, the mutation model described above breaks down for very long alleles, such as those implicated in repeat expansion disorders in humans. Once alleles become long enough, they become unstable and prone to larger and larger expansions. Understanding where and why this phase change occurs will further improve our models of STR mutation.

Finally, it will be important to explore how this model of length-constraint interacts with evolutionary timescales. Models described above were fit on data from timescales spanning modern human history (~50,000 generations). Constraint on allele lengths was also observed when comparing allelic distributions at the same STRs in humans vs. chimps (Garza et al., 1995). Analyzing length constraints across more divergent species becomes challenging. While many homologous STRs can be identified across species, many individual STR loci are species- or clade-specific. Further, even for conserved STRs, sequence context is often highly divergent, which may result in differences in mutation patterns unrelated to the length bias. Future work is needed to more fully explore the biological origins of this trend and the implications when studying patterns of STR variation across the tree of life.

## Supplementary Figures

### Supplementary Figure 1


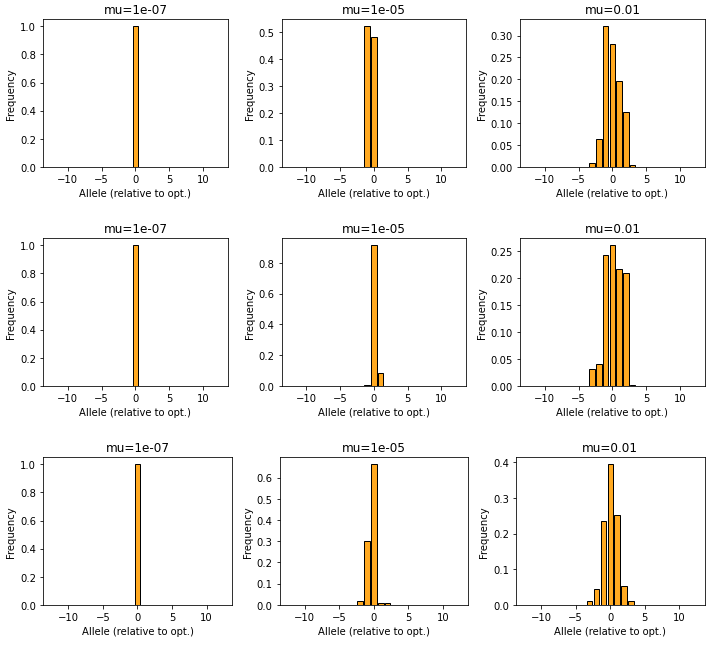


**Example simulated allelic distributions under varying mutation rates.** Each column corresponds to a different mutation rate $\mu$ (from left to right: 1e-7, 1e-5, 0.01). Each row corresponds to a different simulation round. For all panels, other parameters were set to: $\beta$=0.4, $\rho$=0.9, $L$=0.

### Supplementary Figure 2


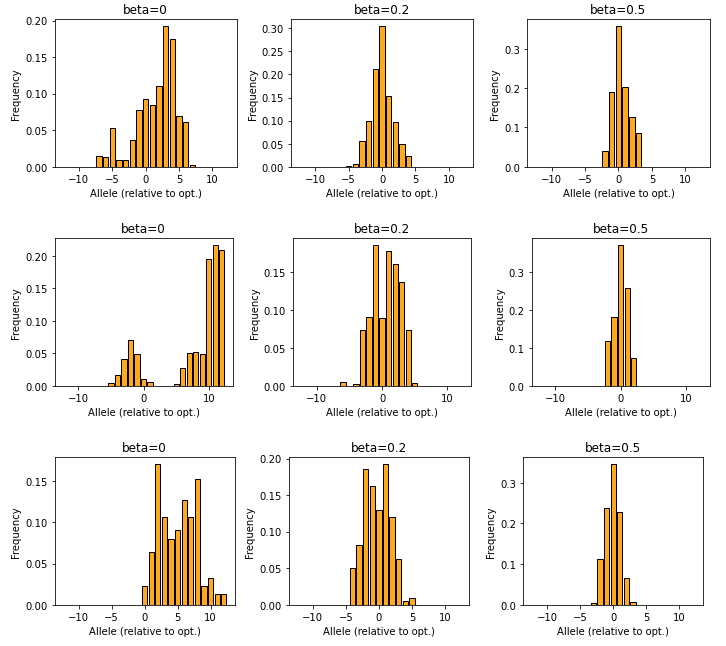


**Example simulated allelic distributions under varying length constraints.** Each column corresponds to a different length constraint ($\beta$) (from left to right: 0, 0.2, 0.5). Each row corresponds to a different simulation round. For all panels, other parameters were set to: $\mu$=0.001, $\rho$=0.9, $L$=0.

### Supplementary Figure 3

**
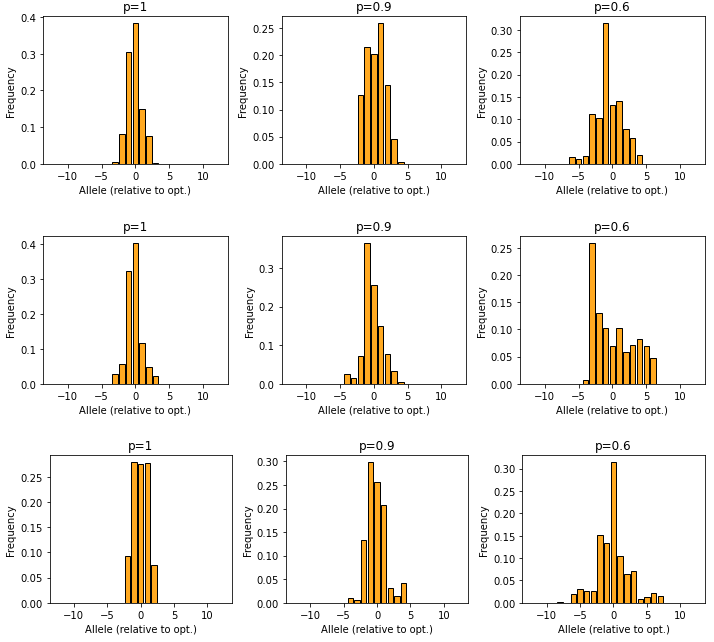
**

**Example simulated allelic distributions under varying values of the step size parameter.** Each column corresponds to a different step size parameter ($\rho$) (from left to right: 1, 0.9, 0.6). Each row corresponds to a different simulation round. For all panels, other parameters were set to: $\mu$=0.001, $\beta$=0.3, $L$=0.

### Supplementary Figure 4

**
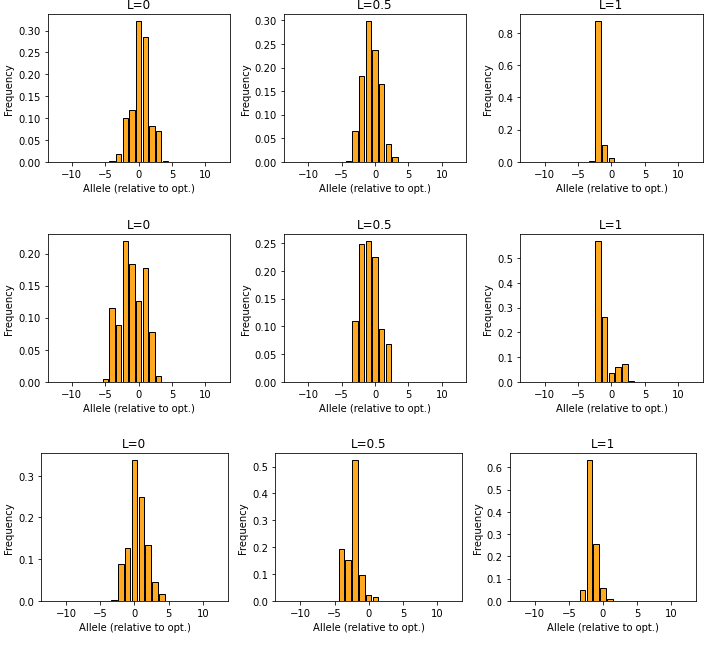
**

**Example simulated allelic distributions under varying mutation rate length-dependence.** Each column corresponds to a different value of $L$ (from left to right: 0, 0.5, 1). Each row corresponds to a different simulation round. For all panels, other parameters were set to: $\mu$=0.001, $\beta$=0.3, $\rho$=0.9.

## Supplementary References

Amos, W., Kosanović, D., & Eriksson, A. (2015). Inter-allelic interactions play a major role in microsatellite evolution. *Proceedings. Biological Sciences / The Royal Society*, *282*(1818), 20152125.

Benson, G. (1999). Tandem repeats finder: a program to analyze DNA sequences. *Nucleic Acids Research*, *27*(2), 573–580.

Bhargava, A., & Fuentes, F. F. (2010). Mutational dynamics of microsatellites. *Molecular Biotechnology*, *44*(3), 250–266.

Garza, J. C., Slatkin, M., & Freimer, N. B. (1995). Microsatellite allele frequencies in humans and chimpanzees, with implications for constraints on allele size. *Molecular Biology and Evolution*, *12*(4), 594–603.

Gymrek, M., Willems, T., Reich, D., & Erlich, Y. (2017). Interpreting short tandem repeat variations in humans using mutational constraint. *Nature Genetics*, *49*(10), 1495–1501.

Haasl, R. J., & Payseur, B. A. (2013). Microsatellites as Targets of Natural Selection. *Molecular Biology and Evolution*, *30*(2), 285–298.

Harr, B., & Schlötterer, C. (2000). Long microsatellite alleles in Drosophila melanogaster have a downward mutation bias and short persistence times, which cause their genome-wide underrepresentation. *Genetics*, *155*(3), 1213–1220.

Heissl, A., Betancourt, A. J., Hermann, P., Povysil, G., Arbeithuber, B., Futschik, A., Ebner, T., & Tiemann-Boege, I. (2019). The impact of poly-A microsatellite heterologies in meiotic recombination. *Life Science Alliance*, *2*(2). https://doi.org/10.26508/lsa.201900364

Kelkar, Y. D., Tyekucheva, S., Chiaromonte, F., & Makova, K. D. (2008). The genome-wide determinants of human and chimpanzee microsatellite evolution. *Genome Research*, *18*(1), 30–38.

Kent, W. J., Sugnet, C. W., Furey, T. S., Roskin, K. M., Pringle, T. H., Zahler, A. M., & Haussler, D. (2002). The human genome browser at UCSC. *Genome Research*, *12*(6), 996–1006.

Mitra, I., Huang, B., Mousavi, N., Ma, N., Lamkin, M., Yanicky, R., Shleizer-Burko, S., Lohmueller, K. E., & Gymrek, M. (2021). Patterns of de novo tandem repeat mutations and their role in autism. *Nature*, *589*(7841), 246–250.

Mousavi, N., Shleizer-Burko, S., Yanicky, R., & Gymrek, M. (2019). Profiling the genome-wide landscape of tandem repeat expansions. *Nucleic Acids Research*, *47*(15), e90.

Payseur, B. A., Jing, P., & Haasl, R. J. (2011). A genomic portrait of human microsatellite variation. *Molecular Biology and Evolution*, *28*(1), 303–312.

Sainudiin, R., Durrett, R. T., Aquadro, C. F., & Nielsen, R. (2004). Microsatellite mutation models: insights from a comparison of humans and chimpanzees. *Genetics*, *168*(1), 383–395.

Schug, M. D., Hutter, C. M., Wetterstrand, K. A., Gaudette, M. S., Mackay, T. F., & Aquadro, C. F. (1998). The mutation rates of di-, tri- and tetranucleotide repeats in Drosophila melanogaster. *Molecular Biology and Evolution*, *15*(12), 1751–1760.

Sun, J. X., Helgason, A., Masson, G., Ebenesersdóttir, S. S., Li, H., Mallick, S., Gnerre, S., Patterson, N., Kong, A., Reich, D., & Stefansson, K. (2012). A direct characterization of human mutation based on microsatellites. *Nature Genetics*, *44*(10), 1161–1165.

Vigouroux, Y., Jaqueth, J. S., Matsuoka, Y., Smith, O. S., Beavis, W. D., Smith, J. S. C., & Doebley, J. (2002). Rate and pattern of mutation at microsatellite loci in maize. *Molecular Biology and Evolution*, *19*(8), 1251–1260.

Wierdl, M., Dominska, M., & Petes, T. D. (1997). Microsatellite instability in yeast: dependence on the length of the microsatellite. *Genetics*, *146*(3), 769–779.

Willems, T., Gymrek, M., Poznik, G. D., Tyler-Smith, C., 1000 Genomes Project Chromosome Y Group, & Erlich, Y. (2016). Population-Scale Sequencing Data Enable Precise Estimates of Y-STR Mutation Rates. *American Journal of Human Genetics*, *98*(5), 919–933.
